# When treatment becomes risk: polypharmacy and inappropriate prescribing in cognitive impairment in 2379 consecutive patients from a memory clinic

**DOI:** 10.1002/alz.71708

**Published:** 2026-08-25

**Authors:** Xavier Morató, Adrian Hinojosa‐Calleja, Sara Jofresa, Berta Calm, Amanda Cano, Josep Blazquez‐Folch, Emilio Alarcón‐Martín, Laura Montrreal, Álvaro Muñoz‐Morales, Claudia Olivé, Paula Bayón, Oscar Sotolongo‐Grau, Adelina Orellana, Pilar Sanz‐Cartagena, Xavier Montalban, Marta Marquié, Maria Victoria Fernandez, Agustín Ruiz, Mercè Boada, Sergi Valero

**Affiliations:** ^1^ Ace Alzheimer Center Barcelona, Universitat Internacional de Catalunya Barcelona Spain; ^2^ Networking Research Center on Neurodegenerative Diseases (CIBERNED) Instituto de Salud Carlos III Madrid Spain; ^3^ Glenn Biggs Institute for Alzheimer's & Neurodegenerative Diseases University of Texas Health Science Center San Antonio Texas USA; ^4^ Department of Microbiology, Immunology and Molecular Genetics, Long School of Medicine University of Texas Health Science Center San Antonio Texas USA

**Keywords:** Alzheimer's disease, cognitive impairment, drug‐induced cognitive impairment, estimated glomerular filtration rate, polypharmacy, potentially inappropriate medications

## Abstract

**BACKGROUND:**

Polypharmacy, potentially inappropriate medications (PIMs), and drugs associated with drug‐induced cognitive impairment (DICI) are highly prevalent in older adults and may affect cognitive decline.

**METHODS:**

This observational retrospective study included 2379 patients evaluated at Ace Alzheimer Center Barcelona. Medication data were extracted from electronic health records using a large language model (LLM). Polypharmacy (≥5), hyperpolypharmacy (≥10), PIMs, and DICI drugs were analyzed across cognitive stages, Alzheimer's disease biomarkers, ApoE subtype, and estimated glomerular filtration rate.

**RESULTS:**

Among patients with cognitive impairment (Clinical Dementia Rating [CDR] ≥ 0.5), 67% presented polypharmacy and 21% hyperpolypharmacy. Higher odds of polypharmacy were observed across the CDR scale, while higher education showed a protective effect. Altered renal function was found in 20.8% of patients with polypharmacy. Indeed, significant differences were found between amyloid beta‐positive and negative (Aβ^+^/Aβ^−^) subjects and between the whole population and subjects participating in clinical trials.

**DISCUSSION:**

Polypharmacy is extremely common in patients attending memory clinics. LLMs could facilitate systematic structured medication reviews.

## Highlights

- In this real‐world cohort of 2379 individuals, 64.4% presented polypharmacy and 19.3% hyperpolypharmacy, rising to 67% among patients with cognitive impairment. Furthermore, 61.6% of patients were exposed to at least one inappropriate medication (PIM or DICI), increasing to 74.5% in polypharmacy and 88.5% in hyperpolypharmacy.
- LLM‐based extraction of medication data enables a scalable and systematic approach to medication review in memory clinic populations. This facilitates the identification of high‐risk prescribing patterns and supports personalized prevention and optimization strategies within multi‐domain care plans.
- Medication burden showed a clear gradient across CDR stages and was significantly associated with older age, while higher educational level had a protective effect.

## 1 BACKGROUND

Population aging has led to a substantial increase in the prevalence of multimorbidity and chronic medication use.^1^ Polypharmacy (PP), defined as the concomitant use of five or more medications, has become the norm rather than the exception in older adults. A systematic review and meta‐analysis of observational studies, defining polypharmacy (≥5 drugs) in adults aged ≥60, included 57 million individuals reporting a global PP prevalence of 39.1% and hyperpolypharmacy (HP) (≥10 drugs) of 13.3%.^2^ This meta‐analysis showed that the prevalence of PP was significantly higher in individuals aged 70 or older in developed regions (i.e., Europe had a 45% PP). While multiple medications are often clinically justified, excessive or inappropriate drug use is associated with a wide range of adverse outcomes, including falls, hospitalization, frailty, and mortality. At the same time, the relationship between cognitive impairment and medication burden is likely bidirectional, suggesting that PP may reflect underlying clinical complexity and disease severity as much as a potential contributor to adverse outcomes.

Physiological aging is accompanied by important pharmacokinetic and pharmacodynamic changes (i.e., reduced renal clearance). The prevalence of chronic kidney disease (CKD) increases with age, with important clinical implications related to the accumulation and altered handling of renally excreted drugs.^3^ At the same time, increased central nervous system (CNS) sensitivity renders older adults particularly vulnerable to medications with sedative, anticholinergic, or psychoactive properties. Due to changes in metabolism and increased sensitivity, a group of drugs has been categorized as potentially inappropriate medications (PIMs) for older adults. PIMs are drugs that carry risks exceeding their potential benefits or for which safer alternatives exist. These issues are especially relevant in patients with cognitive impairment, in whom subtle medication‐related cognitive effects may accelerate decline or mimic neurodegenerative disease. Drug‐induced cognitive impairment (DICI) describes a potentially reversible decline in cognitive function attributable primarily to pharmacological exposure. Both centrally acting and non‐centrally acting drugs may contribute through diverse mechanisms. Several drug classes frequently prescribed in older adults have been associated with DICI, increased risk of dementia, and/or mortality.^4^ Benzodiazepines, related hypnotic and sedative drugs, and opioids are associated with an increased risk of falls and fractures, reduced reflexes, psychiatric adverse reactions, and depressive symptoms, and several studies have demonstrated that their long‐term use can cause impairment in numerous dimensions of cognitive function.^5^, ^6^, ^7^, ^8^ Antidepressants have been associated with sedation, hyponatremia, increased risk of falls and fractures, and potential acceleration of cognitive decline.

In a nationwide Swedish cohort in 18,740 patients, antidepressant treatment was associated with faster cognitive decline compared with non‐users.^9^ In addition, anticholinergic medications impair cholinergic neurotransmission by blocking the action of acetylcholine, essential for memory and learning, thereby contributing to cognitive decline and functional deterioration. In a large meta‐analysis study in older adults with dementia, antipsychotic use was associated with a significantly increased risk of all‐cause mortality, underscoring important safety concerns in routine clinical practice.^10^ Glucocorticoids, antiepileptics, and dopaminergic drugs have also been associated with cognitive impairment.^11^, ^12^, ^13^ In addition, PP itself may act synergistically with underlying Alzheimer's disease (AD).^14^ In parallel, preclinical studies in mouse models of AD have begun to show that the effects of PP are strongly sex‐dependent, underscoring sex as a critical biological variable when evaluating the risks and potential benefits of multi‐drug regimens.^15^

The main objectives of this study were to determine the prevalence of PP, HP PIMs, and DICI in adults with cognitive disorders in a large, real‐world memory clinic cohort. Likewise, we evaluated the associated factors, including sex, age, cognitive status, confirmed AD biomarkers, apolipoprotein E (ApoE) status, and CKD. To ensure accurate medication classification, large language models (LLMs) were employed to support automated extraction and mapping of medications to their corresponding therapeutic codes. Finally, differences in medication use patterns between patients eligible for clinical trials and the typical patient profile seen in routine memory clinic practice were explored to assess whether trial populations adequately reflect real‐world clinical complexity, given that PP may influence trial eligibility.

## 2 METHODS

### 2.1 Study design and ethical approval

This retrospective observational study included 2379 consecutive patients who received a cognitive evaluation for the first time at the Ace Alzheimer Center Barcelona (ACE) between January 2024 and April 2025. ACE is a specialized center providing comprehensive diagnostic evaluation, follow‐up, and research participation for individuals at risk of cognitive impairment and with cognitive impairment and dementia.^16^ All participants involved in the study provided written informed consent before the inclusion of their data in the study, in accordance with the Declaration of Helsinki and applicable national regulations. All consecutive patients undergoing a standardized diagnostic work‐up were eligible for inclusion. Clinical assessment included medical history, neurological examination, neuropsychological testing, and functional evaluation using the Global Clinical Dementia Rating (CDR‐G)^17^ scale and the Mini‐Mental State Examination (MMSE).^18^ A subgroup of participants (*n* = 1008) had available ApoE genotype data, of whom a subsample (*n* = 488) also underwent cerebrospinal fluid (CSF) biomarker analysis for AD, enabling stratification according to amyloid status.^19^ Estimated glomerular filtration rate (eGFR) data were available for a subset of patients included in the study (*n* = 472), allowing renal function to be assessed within this group, which is relevant given its potential impact on drug clearance. The research protocol was approved by the Ethics Committee of the Hospital Universitari de Bellvitge (EOM019/24).

**RESEARCH IN CONTEXT**

**Systematic review**: The authors reviewed the literature using traditional scientific databases and recent publications addressing PP, PIMs, DICI, and medication‐related risks in older adults with cognitive disorders. Previous studies demonstrated associations between PP, adverse clinical outcomes, cognitive decline, and dementia risk, but large real‐world analyses in memory clinic populations remain limited. Furthermore, the use of LLMs for systematic medication extraction and review in cognitive disorders has been scarcely investigated. Relevant studies on anticholinergic medications, benzodiazepines, antidepressants, antipsychotics, CKD, and medication burden in aging populations are appropriately cited.

**Interpretation**: Our findings demonstrate an exceptionally high prevalence of PP, HP, and exposure to potentially inappropriate or cognition‐impairing medications among individuals attending a memory clinic, highlighting medication burden as a major challenge in populations at risk of cognitive decline and dementia. Increasing cognitive severity was associated with greater medication burden and higher exposure to DICI‐associated drugs. Importantly, this study shows that LLM‐based extraction of medication data from electronic health records can provide an accurate, scalable, and reproducible approach to medication review, facilitating the identification of high‐risk prescribing patterns in routine clinical practice.

**Future directions**: Future longitudinal studies should determine whether reducing PP, PIMs, and DICI exposure modifies cognitive trajectories, functional outcomes, and treatment safety in patients with cognitive impairment. Research should evaluate the implementation of LLM‐assisted medication review systems in memory clinics, assess their impact on deprescribing interventions and clinical decision‐making, and investigate how medication burden interacts with AD biomarkers, renal function, and pharmacogenetic factors. Additional studies are needed to understand the influence of PP on the safety, effectiveness, and generalizability of emerging disease‐modifying therapies in real‐world populations.

### 2.2 Medication data extraction, classification, and validation

PP was defined as the concurrent use of five or more medications and HP as 10 or more medications. HP is included within PP estimations, meaning that patients taking 10 or more medications are already counted among those taking five or more medications. Medication data were primarily obtained from the Catalan electronic prescription and dispensing system (SIRE; Sistema Integral de Recepta Electrònica) and complemented by information extracted from free‐text electronic health records using a LLM (Mistral 7B). These free‐text entries originate from clinical notes recorded during routine anamnesis, enabling the identification of medications added by clinicians that are not captured in structured prescription records.^20^ The extraction pipeline identified drug names from both structured and narrative clinical records and standardized them by mapping each medication to its corresponding Anatomical Therapeutic Chemical (ATC) code using the Spanish Medicines Agency CIMA database.

Drugs were classified as potentially inappropriate if they met at least one of the following criteria: (1) inclusion in two or more reference databases (Beers, STOPP/START, EU‐PIM, or PRISCUS),^21^, ^22^, ^23^, ^24^ (2) an explicit recommendation from the summary of product characteristics (SmPC) of a medicinal product, (3) a safety alert issued by the Spanish Agency of Medicines and Medical Devices (AEMPS), or (4) consensus agreement among an expert panel.^25^ Drugs associated with cognitive impairment were defined as medications with evidence of DICI, including anticholinergics (Table S1), benzodiazepines (ATC codes N05B and N05C) and related hypnotic and sedative drugs (N05CF), opioids (N02A), antipsychotics (N05A), selected antidepressants (N06AB), antiepileptics (N03), dopaminergic agents (N04B), and glucocorticoids (H02AB). Finally, any inappropriate medication use was defined as the presence of at least one medication that was either potentially inappropriate for older adults or associated with cognitive impairment.

A random sample of 117 records (5% of the total) was reviewed to quantify extraction performance. The mean absolute error (MAE) in the number of medications per patient was 0.21, with a 95% confidence interval (CI) of 0.00 to 0.49. Agreement between reference and extracted ATC codes was high, with a mean Jaccard similarity of 0.95 (95% CI: 0.90 to 1.00), indicating excellent concordance. DICI classification showed high performance, with an accuracy of 97.44% (95% CI: 92.81 to 99.04), sensitivity of 96.30% (95% CI: 89.67 to 98.73), and specificity of 100.0% (95% CI: 90.36 to 100), corresponding to less than 3% classification error and no false‐positive DICI classifications. PIM classification, corresponding to medications inappropriate for elderly patients, showed no errors in the validation sample, with accuracy, sensitivity, and specificity all equal to 100% (Table S2).

Additionally, the estimated prevalence of selected pharmacological classes in the sample was as follows: DICI use 51.28% (95% CI: 42.45 to 60.11), benzodiazepine use 33.33% (95% CI: 25.00 to 41.66), antipsychotic use 13.68% (95% CI: 7.60 to 19.75), hypnotic use 1.71% (95% CI: 0 to 4.00), opioid use 12.82% (95% CI: 6.91 to 18.73), antidepressant use 28.21% (95% CI: 20.25 to 36.16), antiepileptic use 3.42% (95% CI: 0.21 to 6.63), dopaminergic use 3.42% (95% CI: 0.21 to 6.63), glucocorticoid use 1.71% (95% CI: 0.00 to 4.00), and anticholinergic use 46.15% (95% CI: 37.35 to 54.96). CIs were computed using a normal approximation with finite population correction to account for sampling without replacement from the total population. Notably, all corresponding prevalence estimates reported in Table 6 fall within these CIs, providing strong empirical support for the accuracy and reliability of the ATC code extraction pipeline.

### 2.3 Statistical analysis

Descriptive statistics were used to summarize demographic, clinical, and pharmacological characteristics. Associations between demographic and clinical factors and medication burden were evaluated using multivariable logistic regression models, considering PP (≥5 medications) and PP (≥10 medications) as binary outcomes. Age was dichotomized at 75 years (≥75 vs < 75), sex was coded as female versus male, and years of schooling and MMSE score were included as continuous variables. CDR‐G was modeled as a categorical variable and grouped as 0, 0.5, 1, and ≥2, with CDR = 0 used as the reference category. Odds ratios (ORs) and 95% CIs were estimated. This model was defined as the primary analytical framework based on clinical interpretability, parsimony, and robustness across outcomes.

To assess whether genetic risk modified the associations observed in the primary analyses, additional multivariable logistic regression models were fitted in the subsample of participants with available ApoE genotyping (*n* = 1008). ApoE status was modeled as a binary variable, contrasting ε4 carriers (≥1 ε4 allele) with non‐carriers, and was added to the primary model as an additional covariate. All other predictors were specified identically to the main analysis. Collinearity among predictors included in the primary model was assessed as part of model diagnostics using variance inflation factors (VIFs). This procedure was applied to verify the stability of regression estimates and support appropriate model specifications. Potential effect modification was explored by testing first‐order interaction terms between demographic and clinical predictors included in the primary model. Interactions were evaluated in an exploratory manner by adding one interaction term at a time to the fully adjusted main‐effects model, which always retained all primary predictors. Models including multiple interaction terms simultaneously were not fitted to avoid overparameterization, preserve statistical power, and maintain interpretability, particularly given the categorical specification of CDR and the lower event rate for HP. Interaction terms were retained only if they demonstrated consistent statistical significance and improved model fit.

In addition to regression‐based analyses, descriptive comparisons of the prevalence of specific medication classes (anticholinergics, benzodiazepines, antipsychotics, opioids, and antidepressants) were conducted across medication burden categories (<5 medications, PP, and HP) in the overall cohort and stratified by age group (<75 and ≥75 years), as well as within predefined subsamples according to AD biomarker status (Aβ positive vs Aβ negative in CSF subsample), clinical trial participation status (CT vs non‐CT), and CKD (G1 ≥90, G2 60 to 89, G3a 45 to 59, G3b 30 to 44, G4 15 to 29 mL/min/1.73 m^2^) stage, defined according to Kidney Disease | Improving Global Outcomes (KDIGO) guidelines. Frequencies and percentages were calculated for each subgroup, and between‐group differences were evaluated using Pearson's chi‐squared tests. Statistical significance was assessed using two‐sided tests with a significance level of 0.05. All analyses were performed using STATA version 15.

## 3 RESULTS

### 3.1 Sample characteristics, PP, and HP

**FIGURE 1:**
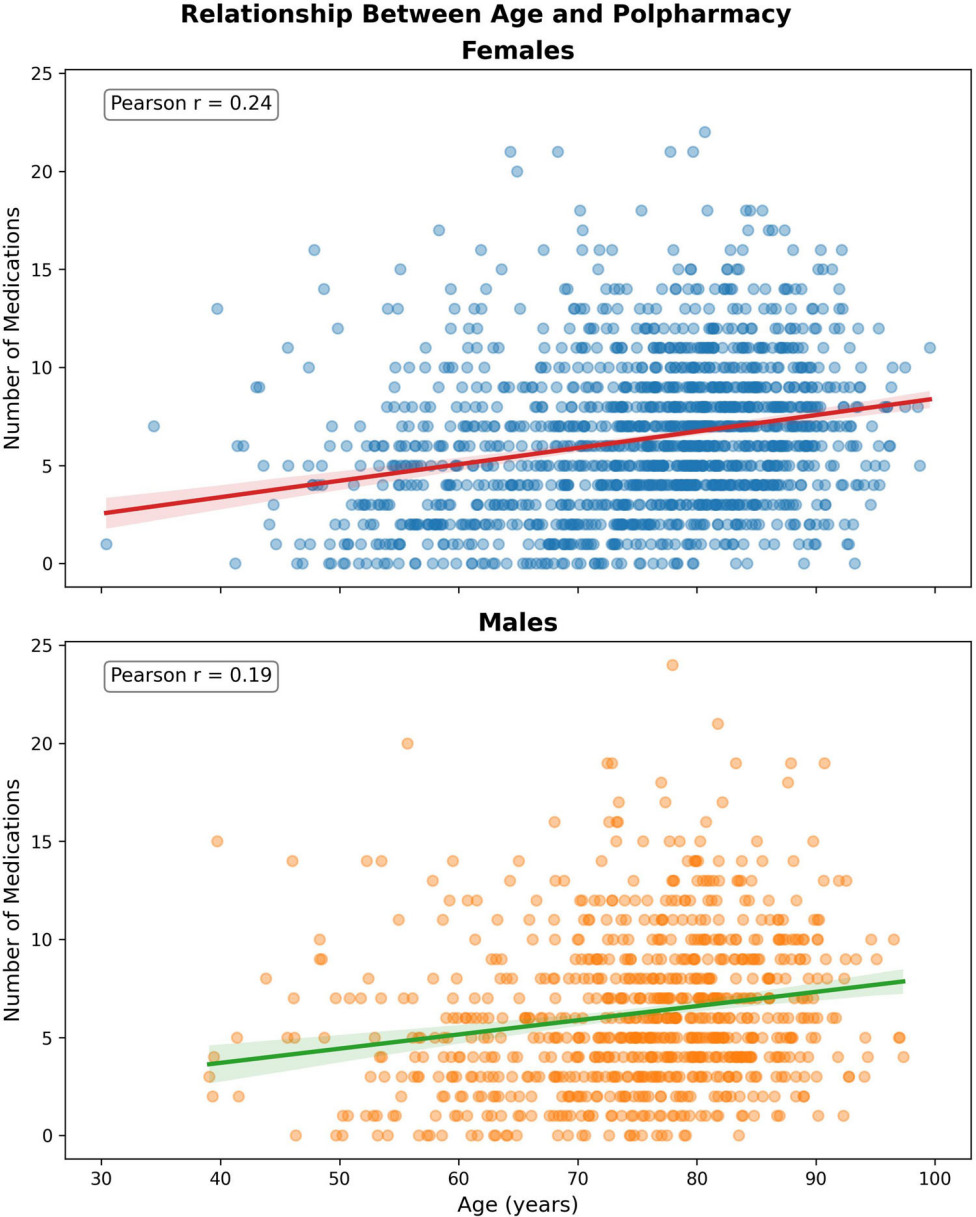
Relationship between age and polypharmacy in males and females.

**FIGURE 2:**
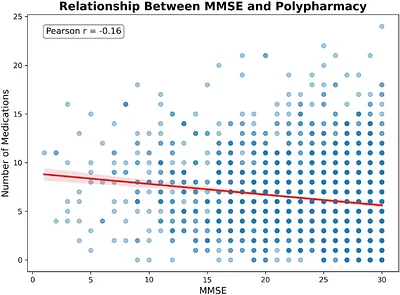
Relationship between Mini‐Mental State Examination score and polypharmacy.

The study included 2379 participants (mean age 75.4 ± 10.9 years), of whom 64.7% were women, with a mean medication burden of 6.3 ± 3.9 drugs. Overall, 64.4% of participants met criteria for PP (≥5 medications) and 19.3% for HP (≥10 medications). Both PP and HP were more prevalent in older participants, particularly those aged ≥75 years, among whom 72.3% had PP and 22.3% HP, compared with 52.8% and 14.9%, respectively, in those aged <75 years (Table 1). Age was positively associated with the number of medications in both sexes, indicating increasing PP with advancing age. This relationship was modest but statistically meaningful, with a slightly stronger correlation observed in women (Pearson *r* = 0.24) than in men (Pearson *r* = 0.19) (Figure 1). Among patients with cognitive impairment (CDR 0.5 to 3; *n* = 2103), 67.1% (*n* = 1410) were exposed to PP (≥5 medications), and 20.5% (*n* = 430) met criteria for HP (≥10 medications) (Table S3). For PP (≥5 medications), older age (≥75 years) was significantly associated with higher odds of PP (OR = 1.70, 95% CI: 1.39 to 2.07; *p* < 0.001) (Table 2). An inverse association between global cognitive performance, measured by the MMSE, and medication burden was observed. Higher numbers of prescribed medications were modestly associated with lower MMSE scores (Pearson *r* = −0.16), indicating that greater levels of PP tend to occur in individuals with poorer cognitive performance (Figure 2). A clear gradient was observed across CDR categories, with progressively higher odds compared with cognitively unimpaired participants (CDR = 0): OR = 1.66 (95% CI: 1.23 to 2.25; *p* = 0.001) for CDR 0.5, OR = 2.11 (95% CI: 1.45 to 3.08; *p* < 0.001) for CDR 1, and OR = 2.70 (95% CI: 1.59 to 4.57; *p* < 0.001) for CDR ≥2. Years of schooling showed a significant inverse association with PP (OR = 0.96 per additional year, 95% CI: 0.94 to 0.98; *p* < 0.001). Sex and MMSE score were not significantly associated with PP (Table 2). For HP (≥10 medications), CDR severity remained the strongest determinant, with significantly increased odds across all impairment levels compared with CDR = 0 (OR = 1.82, 95% CI: 1.11 to 2.99; *p* = 0.018 for CDR 0.5; OR = 2.61, 95% CI: 1.51 to 4.52; *p* < 0.001 for CDR 1; and OR = 4.10, 95% CI: 2.10 to 8.00; *p* < 0.001 for CDR ≥2). In contrast to PP, age ≥75 years was not independently associated with HP. Higher educational attainment remained protective (OR = 0.95 per year, 95% CI: 0.92 to 0.97; *p* < 0.001), while MMSE score showed a modest but significant association with HP (OR = 1.04 per point, 95% CI: 1.00 to 1.07; *p* = 0.026). Sex was not associated with HP.

**TABLE 1:** Demographics: whole sample description.

Group	Sample size (%)	Medication (mean ± std)	Sex (*n* females, % females)	Age (mean ± SD)
**Whole population**				
Whole sample size	2379 (100.0)	6.32 ± 3.87	1540 (64.7)	75.40 ± 10.85
<5 medications	847 (35.6)	2.46 ± 1.33	537 (63.4)	72.27 ± 11.33
Polypharmacy (≥5 meds)	1532 (64.4)	8.45 ± 3.09	1003 (65.5)	77.13 ± 10.17
Hyperpolypharmacy (≥10 meds)	459 (19.3)	12.31 ± 2.45	295 (64.3)	77.88 ± 9.95
**Age < 75**				
Whole sample size	969 (100.0)	5.38 ± 3.96	616 (63.6)	64.88 ± 8.15
<5 medications	457 (47.2)	2.13 ± 1.34	297 (65.0)	63.92 ± 8.17
Polypharmacy (≥5 meds)	512 (52.8)	8.28 ± 3.20	319 (62.3)	65.74 ± 8.04
Hyperpolypharmacy (≥10 meds)	144 (14.9)	12.56 ± 2.50	90 (62.5)	66.14 ± 7.90
**Age ≥ 75**				
Whole sample size	1410 (100.0)	6.96 ± 3.68	924 (65.5)	82.63 ± 4.95
<5 medications	390 (27.7)	2.84 ± 1.21	239 (61.5)	82.04 ± 4.81
Polypharmacy (≥5 medications)	1020 (72.3)	8.53 ± 3.02	684 (67.1)	82.85 ± 4.99
Hyperpolypharmacy (≥10 meds)	315 (22.3)	12.20 ± 2.43	205 (65.1)	83.24 ± 4.89

**TABLE 2:** Multivariable logistic regression models from the whole population, APOE and CSF Aβ42/40 subsamples.

Multivariable logistic regression: predictors of polypharmacy and hyperpolypharmacy												
Predictor	Full cohort (*n* = 2379)				APOE ε4 subsample (*n* = 1008)				CSF Aβ40/42 subsample (*n* = 472)			
	Polypharmacy (≥5)		Hyperpolypharmacy (≥10)		Polypharmacy (≥5)		Hyperpolypharmacy (≥10)		Polypharmacy (≥5)		Hyperpolypharmacy (≥10)	
	OR (95% CI)	*P* value	OR (95% CI)	*P* value	OR (95% CI)	*P* value	OR (95% CI)	*P* value	OR (95% CI)	*P* value	OR (95% CI)	*P* value
Age ≥75 years	1.70 (1.39, 2.07)	**<0.001**	1.12 (0.87, 1.44)	0.38	1.58 (1.14, 2.21)	**0.007**	0.89 (0.58, 1.36)	0.59	1.79 (1.20, 2.67)	**0.004**	0.79 (0.43, 1.46)	0.457
CDR 0.5 versus 0	1.66 (1.23, 2.25)	**0.001**	1.82 (1.11, 2.99)	**0.018**	2.56 (1.57, 4.20)	**<0.001**	1.84 (0.84, 4.02)	0.13	—	—	—	—
CDR 1 versus 0	2.11 (1.45, 3.08)	**<0.001**	2.61 (1.51, 4.52)	**<0.001**	2.57 (1.38, 4.79)	**0.003**	2.68 (1.10, 6.51)	**0.030**	—	—	—	—
CDR 1 versus 0.5	—	—	—	—	—	—	—	—	0.99 (0.62, 1.59)	0.972	1.66 (0.83, 3.30)	0.150
CDR ≥2 versus 0	2.70 (1.59, 4.57)	**<0.001**	4.10 (2.10, 8.00)	**<0.001**	3.25 (1.31, 8.07)	**0.011**	3.59 (1.13, 11.38)	**0.030**	—	—	—	—
Female sex	1.00 (0.83, 1.20)	0.97	0.92 (0.73, 1.15)	0.45	0.85 (0.62, 1.17)	0.32	0.86 (0.58, 1.27)	0.44	1.20 (0.81, 1.76)	0.367	1.24 (0.68, 2.26)	0.477
Years of schooling	0.96 (0.94, 0.98)	**<0.001**	0.95 (0.92, 0.97)	**<0.001**	0.98 (0.95, 1.02)	0.34	0.95 (0.91, 1.00)	**0.047**	0.96 (0.92, 1.00)	0.076	0.90 (0.84, 0.98)	**0.012**
MMSE score	1.02 (0.99, 1.04)	0.28	1.04 (1.00, 1.07)	**0.026**	1.00 (0.96, 1.05)	0.92	1.03 (0.97, 1.08)	0.37	1.00 (0.93, 1.07)	0.906	1.09 (0.98, 1.21)	0.099
APOE ε4 carrier	—	—	—	—	0.86 (0.62, 1.19)	0.37	0.72 (0.47, 1.11)	0.14	—	—	—	—
CSF Aβ40/42 positivity (1 vs 0)	—	—	—	—	—	—	—	—	0.72 (0.48, 1.07)	0.106	0.80 (0.44, 1.47)	0.474

*Note*: Outcomes: polypharmacy (≥5 drugs) and hyperpolypharmacy (≥10 drugs). Bold *p* values indicate statistical significance (*p* < 0.05). — = predictor not included in model.

Full cohort — reference categories: age < 75 years; Clinical Dementia Rating (CDR) = 0; male sex. APOE subsample – reference categories: age < 75 years; CDR = 0; male sex; no APOE ε4; *n* = 1008. CSF subsample – CSF Aβ40/42 subsample; CDR reference category: CDR 0.5 (CDR 1 vs 0.5).

To further assess medication burden as a continuous trait, we fitted an ordinal logistic regression model with CDR severity as the ordered outcome. The model included total number of medications, DICI burden, age, sex, and years of education. Higher medication count was independently associated with greater CDR severity (OR = 1.03, 95% CI: 1.01 to 1.04; *p* < 0.001), as was higher DICI burden (OR = 1.04, 95% CI: 1.00 to 1.08; *p* = 0.039). Age was also positively associated with CDR severity (OR = 1.06, 95% CI: 1.05 to 1.07; *p* < 0.001), whereas years of education showed an inverse association (OR = 0.94, 95% CI: 0.93 to 0.95; *p* < 0.001). Sex was not significantly associated with CDR severity after adjustment for the other covariates (OR = 0.92, 95% CI: 0.84 to 1.02; *p* = 0.107).

In the ApoE subsample (Table 2), the main associations observed in the total cohort were largely preserved. For PP (≥5 medications), older age (≥75 years; OR = 1.58, *p* = 0.007) and increasing CDR severity (e.g., CDR ≥ 2 vs 0: OR = 3.25, *p* = 0.011) remained independently associated with higher odds, while ApoE ε4 carrier status was not associated (*p* > 0.30). For HP (≥10 medications), CDR severity was the dominant determinant (CDR ≥ 2 vs 0: OR = 3.59, *p* = 0.030), whereas age was no longer associated (*p* = 0.59). Across both outcomes, educational attainment showed a modest protective effect (OR ≈ 0.95 per year, *p* ≤ 0.05).

To evaluate the stability of the regression estimates, collinearity among predictors included in the primary model was assessed as part of model diagnostics. Moderate collinearity was observed, primarily between CDR and MMSE, as expected given their overlapping clinical constructs. VIFs remained below commonly used thresholds for problematic collinearity (maximum VIF 3.2 to 4.6 for CDR categories and 2.9 for MMSE, with all other predictors <1.5), indicating that collinearity did not materially affect the robustness or interpretability of the model results.^26^ Exploratory analyses testing first‐order interactions between demographic and clinical predictors did not identify any interaction that consistently improved model fit or showed a robust association with either PP or HP. The effects of CDR, age, and education were largely additive, supporting the use of the main‐effects model as the primary analytical framework.

In the CSF subsample, analyses restricted to participants with CDR 0.5 or 1 (*n* = 479), 53.7% of participants were biomarker‐positive. CSF Aβ40/42 positivity (defined as Aβ 42/40 ≤0.063 pg/mL) was not independently associated with either PP or HP after multivariable adjustment (Table 2).

In the subsample or participants with kidney function information (eGFR), most individuals were classified in G2 (55.1%), followed by G1 (28.5%), while more severe groups (G3A, G3B, G4) were progressively less frequent (12.6%, 3.3%, and 0.5%, respectively) (Table 3). Age‐stratified analyses revealed marked differences. Among participants aged <75 years, the distribution was concentrated in G1–G2 (94.8%), with very low representation in more severe groups (G3A–G4: 5.3%). In contrast, participants aged ≥75 years showed a clear shift toward more severe classifications. Only 14.9% were in G1, while G3A–G4 accounted for 27.3% overall. This gradient intensified with increasing medication burden: In HP, G1 dropped to 7.9%, while G3A–G4 rose to 34.3%, including the highest proportions observed for G3B (7.9%) and G4 (5.3%).

**TABLE 3:** Distribution of participants by estimated glomerular filtration rate, age, and polypharmacy status.

Group	Sample size	G1	G2	G3A	G3B	G4
**Whole population**						
Whole sample size	572	163 (28.5)	315 (55.1)	72 (12.6)	19 (3.3)	3 (0.5)
<5 medications	241	83 (34.4)	133 (55.2)	21 (8.7)	4 (1.7)	0 (0.0)
Polypharmacy (≥5 meds)	331	80 (24.2)	182 (55.0)	51 (15.4)	15 (4.5)	3 (0.9)
Hyperpolypharmacy (≥10 medications)	69	13 (18.8)	38 (55.1)	13 (18.8)	3 (4.3)	2 (2.9)
**Age < 75**						
Whole sample size	284	120 (42.3)	149 (52.5)	14 (4.9)	1 (0.4)	0 (0.0)
<5 medications	141	64 (45.4)	74 (52.5)	3 (2.1)	0 (0.0)	0 (0.0)
Polypharmacy (≥5 medications)	143	56 (39.2)	75 (52.4)	11 (7.7)	1 (0.7)	0 (0.0)
Hyperpolypharmacy (≥10 medications)	31	10 (32.3)	16 (51.6)	5 (16.1)	0 (0.0)	0 (0.0)
**Age ≥ 75**						
Whole sample size	288	43 (14.9)	166 (57.6)	58 (20.1)	18 (6.2)	3 (1.0)
<5 medications	100	19 (19.0)	59 (59.0)	18 (18.0)	4 (4.0)	0 (0.0)
Polypharmacy (≥5 medications)	188	24 (12.8)	107 (56.9)	40 (21.3)	14 (7.4)	3 (1.6)
Hyperpolypharmacy (≥10 medications)	38	3 (7.9)	22 (57.9)	8 (21.1)	3 (7.9)	2 (5.3)

### 3.2 PIMs and DICI drugs

In the overall cohort (*n* = 2379), potentially inappropriate medication use was common, with 20.5% of participants receiving at least one medication considered inappropriate for older adults and 48.3% receiving at least one medication associated with cognitive impairment (Table 4). Overall, 61.6% of participants were exposed to at least one inappropriate medication (including PIMs or DICI). The prevalence of any inappropriate medication use increased markedly with medication burden, rising from 38.5% among individuals taking fewer than five medications to 74.5% in those with PP and 88.5% in those with HP. This dose–response pattern was observed across age strata. Among participants aged < 75 years, any inappropriate medication use affected 39.2% of those taking fewer than five medications, compared with 78.3% and 91.7% in the PP and HP groups, respectively. Similarly, in participants aged ≥75 years, exposure to any inappropriate medication increased from 37.7% in those taking fewer than five medications to 72.6% with PP and 87.0% with HP, indicating a consistent association between increasing medication burden and inappropriate medication exposure across age groups (Table 4). While the majority of participants had one or two DICI drugs, higher counts were predominantly observed among individuals with PP and HP. In the whole population, 21.4% of participants were exposed to one DICI drug and 22.2% to two, whereas exposure to three or more inappropriate medications was uncommon overall but increased substantially with higher medication burden. Among participants with HP, 26.8% had three DICI drugs and 9.4% had four, compared with 4.1% and 0.6%, respectively, among those taking fewer than five medications. These findings indicate a strong accumulation of inappropriate medications as overall medication burden increases, regardless of age (Table S4). Anticholinergic medications were prescribed in 43% of patients, while benzodiazepines were used in 29% (Table 5). Commonly prescribed agents include quetiapine, trazodone, citalopram, diazepam, lorazepam, lormetazepam, and alprazolam (Table S5).

**TABLE 4:** Prevalence of potentially inappropriate medications and drugs related to drug‐induced cognitive impairment.

Group	Sample size	Inappropriate for elderly (*n*, %)	Associated with cognitive impairment (*n*, %)	Any inappropriate (*n*, %)
**Whole population**				
Whole sample size	2379	488 (20.5)	1150 (48.3)	1468 (61.6)
<5 medications	847	86 (10.2)	229 (27.0)	326 (38.5)
Polypharmacy (≥5 medications)	1532	402 (26.2)	921 (60.1)	1142 (74.5)
Hyperpolypharmacy (≥10 medications)	459	163 (35.5)	349 (76.0)	406 (88.5)
**Age < 75**				
Whole sample size	969	260 (26.8)	478 (49.3)	580 (59.9)
<5 medications	457	60 (13.1)	129 (28.2)	179 (39.2)
Polypharmacy (≥5 medications)	512	200 (39.1)	349 (68.2)	401 (78.3)
Hyperpolypharmacy (≥10 medications)	144	73 (50.7)	122 (84.7)	132 (91.7)
**Age ≥ 75**				
Whole sample size	1410	228 (16.2)	672 (47.7)	888 (63.0)
<5 medications	390	26 (6.7)	100 (25.6)	147 (37.7)
Polypharmacy (≥5 medications)	1020	202 (19.8)	572 (56.1)	741 (72.6)
Hyperpolypharmacy (≥10 medications)	315	90 (28.6)	227 (72.1)	274 (87.0)

**TABLE 5:** Prevalence of DICI drugs in the whole, CSF subsample, and clinical trial participants.

CT versus non‐CT subsample	Sample size	DICI (*n*, %)	Benzodiazepine (*n*, %)	Antipsychotic (*n*, %)	Hypnotic (*n*, %)	Opioids (*n*, %)	Antidepressants (*n*, %)	Antiepileptic (*n*, %)	Dopaminergic (*n*, %)	Glucocorticoids (*n*, %)	Anticholinergics (*n*, %)
**Whole population**											
Whole sample size	2379	1489 (62.6)	698 (29.3)	333 (14.0)	45 (1.9)	250 (10.5)	579 (24.3)	131 (5.5)	56 (2.4)	86 (3.6)	1029 (43.3)
<5 medications	847	330 (39.0)	124 (14.6)	52 (6.1)	13 (1.5)	29 (3.4)	133 (15.7)	24 (2.8)	6 (0.7)	15 (1.8)	200 (23.6)
Polypharmacy	1532	1159 (75.7)	574 (37.5)	281 (18.3)	32 (2.1)	221 (14.4)	446 (29.1)	107 (7.0)	50 (3.3)	71 (4.6)	829 (54.1)
Hyperpolypharmacy	459	212 (46.2)	212 (46.2)	122 (26.6)	15 (3.3)	120 (26.1)	155 (33.8)	57 (12.4)	23 (5.0)	38 (8.3)	326 (71.0)
**Age < 75**											
Whole sample size	969	591 (61.0)	292 (30.1)	112 (11.6)	24 (2.5)	114 (11.8)	235 (24.3)	80 (8.3)	17 (1.8)	40 (4.1)	430 (44.4)
<5 medications	457	182 (39.8)	76 (16.6)	23 (5.0)	9 (2.0)	24 (5.3)	79 (17.3)	22 (4.8)	1 (0.2)	14 (3.1)	110 (24.1)
Polypharmacy	512	409 (79.9)	216 (42.2)	89 (17.4)	15 (2.9)	90 (17.6)	156 (30.5)	58 (11.3)	16 (3.1)	26 (5.1)	360 (62.5)
Hyperpolypharmacy	144	133 (92.4)	78 (54.2)	39 (27.1)	7 (4.9)	47 (32.6)	49 (34.0)	31 (21.5)	8 (5.6)	15 (10.4)	117 (81.2)
**Age ≥ 75**											
Whole sample size	1410	898 (63.7)	406 (28.8)	221 (15.7)	21 (1.5)	136 (9.6)	344 (24.4)	51 (3.6)	39 (2.8)	46 (3.3)	599 (42.5)
<5 medications	390	148 (37.9)	48 (12.3)	29 (7.4)	4 (1.0)	5 (1.3)	54 (13.8)	2 (0.5)	5 (1.3)	1 (0.3)	90 (23.1)
Polypharmacy	1020	750 (73.5)	358 (35.1)	192 (18.8)	17 (1.7)	131 (12.8)	290 (28.4)	49 (4.8)	34 (3.3)	45 (4.4)	509 (49.9)
Hyperpolypharmacy	315	277 (87.9)	134 (42.5)	83 (26.3)	8 (2.5)	73 (23.2)	106 (33.7)	26 (8.3)	15 (4.8)	23 (7.3)	209 (66.3)

CSF subsample	Sample size	DICI (*n*, %)	Benzodiazepine (*n*, %)	Antipsychotic (*n*, %)	Hypnotic (*n*, %)	Opioids (*n*, %)	Antidepressants (*n*, %)	Antiepileptic (*n*, %)	Dopaminergic (*n*, %)	Glucocorticoids (*n*, %)	Anticholinergics (*n*, %)
**Whole population**											
Aβ positive	262	146 (55.7)	64 (24.4)	17 (6.5)	5 (1.9)	19 (7.3)	70 (26.7)	8 (3.1)	3 (1.1)	5 (1.9)	89 (34.0)
Aβ negative	226	147 (65.0)	74 (32.7)	24 (10.6)	4 (1.8)	30 (13.3)	59 (26.1)	18 (8.0)	3 (1.3)	7 (3.1)	106 (46.9)
	*p* value	**0.036**	**0.042**	0.100	0.909	**0.027**	0.879	**0.016**	0.855	0.397	**0.004**
**Age < 75**											
Aβ positive	95	50 (52.6)	24 (25.3)	4 (4.2)	1 (1.1)	7 (7.4)	29 (30.5)	5 (5.3)	0 (0.0)	3 (3.2)	26 (27.4)
Aβ negative	145	95 (65.5)	55 (37.9)	18 (12.4)	3 (2.1)	22 (15.2)	41 (28.3)	14 (9.7)	1 (0.7)	4 (2.8)	71 (49.0)
	*p* value	**0.046**	**0.041**	**0.031**	0.548	0.070	0.708	0.2178	0.4173	0.8573	**0.001**
**Age ≥ 75**											
Aβ positive	167	96 (57.5)	40 (24.0)	13 (7.8)	4 (2.4)	12 (7.2)	41 (24.6)	3 (1.8)	3 (1.8)	2 (1.2)	63 (37.7)
Aβ negative	81	52 (64.2)	19 (23.5)	6 (7.4)	1 (1.2)	8 (9.9)	18 (22.2)	4 (4.9)	2 (2.5)	3 (3.7)	35 (43.2)
	*p* value	0.312	0.932	0.917	0.5419	0.466	0.686	0.1612	0.7237	0.1879	0.407
**Whole population**											
CT	82	40 (48.8)	16 (19.5)	6 (7.3)	2 (2.4)	3 (3.7)	19 (23.2)	2 (2.4)	1 (1.2)	0 (0.0)	24 (29.3)
Non‐CT	2297	1449 (63.1)	682 (29.7)	327 (14.2)	43 (1.9)	247 (10.8)	560 (24.4)	129 (5.6)	55 (2.4)	86 (3.7)	1005 (43.8)
*p*‐value		**0.0085**	**0.0467**	0.0760	0.7111	**0.0395**	0.8021	0.2152	0.4905	0.0743	**0.009**
**Age < 75**											
CT	52	24 (46.2)	9 (17.3)	2 (3.8)	1 (1.9)	1 (1.9)	13 (25.0)	2 (3.8)	0 (0.0)	0 (0.0)	14 (26.9)
Non‐CT	917	567 (61.8)	283 (30.9)	110 (12.0)	23 (2.5)	113 (12.3)	222 (24.2)	78 (8.5)	17 (1.9)	40 (4.4)	416 (45.4)
*P* value		**0.0241**	**0.0383**	0.0738	0.7917	**0.0236**	0.8970	0.2349	0.3219	0.1240	**0.009**
**Age ≥ 75**											
CT	30	16 (53.3)	7 (23.3)	4 (13.3)	1 (3.3)	2 (6.7)	6 (20.0)	0 (0.0)	1 (3.3)	0 (0.0)	10 (33.3)
Non‐CT	1380	882 (63.9)	399 (28.9)	217 (15.7)	20 (1.4)	134 (9.7)	338 (24.5)	51 (3.7)	38 (2.8)	46 (3.3)	589 (42.7)
*P* value		0.2332	0.5043	0.7215	0.3993	0.5764	0.5708	0.2835	0.8481	0.3093	0.305

To further evaluate the cumulative exposure to drugs associated with cognitive impairment, we defined the “DICI burden” variable as the number of DICI medication categories used by each participant. DICI burden increased progressively across CDR categories in the whole cohort, from 1.1 ± 1.4 in CDR 0 to 1.9 ± 1.3 in CDR 3, showing a positive correlation with cognitive severity (Spearman ρ= 0.124, *p* < 0.001). Similar patterns were observed in participants aged <75 years and ≥75 years. Analysis of individual medication classes showed that anticholinergic and antipsychotic exposure increased most consistently with CDR severity, whereas benzodiazepine use remained relatively stable, and hypnotic drug use was infrequent. These findings suggest that DICI exposure is not only common in this population but also accumulates with increasing cognitive severity, particularly through specific pharmacological classes (Table S6).

**FIGURE 3:**
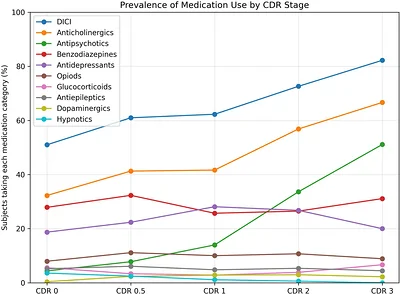
Prevalence of medication use by Clinical Dementia Rating stage.

The class‐specific distribution of medication uses across CDR stages suggested that the overall increase in DICI exposure was mainly driven by anticholinergic and antipsychotic medications, which showed the clearest gradients with increasing cognitive severity (Figure 3). To evaluate this pattern analytically, we fitted ordinal logistic regression models adjusted for age, sex, and years of education (Table S7). Model 1 included overall DICI burden and showed an association with higher CDR severity. Model 2 used a modified DICI burden score excluding anticholinergic and antipsychotic categories; in this model, the association was attenuated and no longer statistically significant. Model 3 included only the combined anticholinergic and antipsychotic burden, which remained associated with CDR severity and showed the best model fit according to the Akaike information criterion and the Bayesian Information Criterion. Together, these findings suggest that the relationship between DICI burden and cognitive severity is not uniformly explained by all DICI medication categories but is mainly driven by anticholinergic and antipsychotic exposure.

In the CSF subsample, comprising 262 Aβ‐positive and 226 Aβ‐negative individuals, differences were observed in the distribution of specific drug categories between groups. Among Aβ‐positive participants, the prevalence of anticholinergic drugs was 34.0% (*n* = 89), antidepressants 26.7% (*n* = 70), antipsychotics 6.5% (*n* = 17), and benzodiazepines 24.4% (*n* = 64). In the Aβ‐negative group, the corresponding prevalence was 46.9% (*n* = 106) for anticholinergic drugs, 26.1% (*n* = 59) for antidepressants, 10.6% (*n* = 24) for antipsychotics, and 32.7% (*n* = 74) for benzodiazepines. Compared with Aβ‐negative individuals, Aβ‐positive participants showed significantly lower use of anticholinergic drugs (*p* = 0.004), opioids (*p* = 0.027), antiepileptics (*p* = 0.016), and benzodiazepines (*p* = 0.042). No statistically significant differences were observed for antipsychotics (*p* = 0.100) or other psychotropic medications (*p* = 0.879) (Table 5). These differences were particularly evident among participants younger than 75 years. In contrast, among participants aged ≥75 years, medication profiles were largely comparable between etiological groups, suggesting that age‐related multimorbidity and generalized PP may attenuate subtype‐specific differences in older populations. We performed additional logistic regression analyses for each medication group, using medication exposure as the dependent variable and Aβ status as the main predictor. Models were adjusted for age, sex, and years of education. While unadjusted analyses suggested differences in several medication classes between amyloid groups, these associations were largely attenuated after adjustment for age, sex, and education. Only DICI and anticholinergic exposure remained significantly more frequent among Aβ‐negative participants, indicating broadly similar medication profiles across amyloid‐defined etiological subgroups (Table S8).

### 3.3 Clinical trial candidates

From the overall sample (*n* = 2379 participants), 82 had been included in AD clinical trials with pharmacological intervention (i.e., anti‐Aβ or anti‐tau monoclonal antibodies, small molecules). Subjects that were eligible for clinical trials (CTs) had a significantly lower mean number of medications compared with the general population (3.87 ± 2.97 vs 6.62 ± 4.10; *p* < 0.001), and they were also significantly younger (71.60 ± 6.85 vs 75.54 ± 10.94 years; *p* < 0.001). The proportion of women was similar between groups (61.0% in CT vs 64.9% in non‐CT), with no statistically significant difference (*p* = 0.543). Overall, CT participants were younger and exposed to a lower medication burden, while sex distribution did not differ between groups (Table 6). In relation with the DICI medication, the prevalence of anticholinergic drug use was significantly lower in the CT group compared with the non‐CT group (29.3% vs 43.8%; *p* = 0.009). Similarly, benzodiazepine use (19.5% vs 29.7%; *p* = 0.046) and opioid use (3.7% vs 10.8%; *p* = 0.040) were significantly less frequent in the CT group. Although antipsychotic use was also lower in the CT group (7.3% vs 14.2%), this difference did not reach statistical significance (*p* = 0.076). No differences were observed in antidepressant use between groups (23.2% vs 24.4%; *p* = 0.802) (Table 5).

**TABLE 6:** Clinical trial–eligible participants compared with overall cohort.

Group	Variable	Non‐CT	CT	T	*p* value
Whole sample size	Sample size (%)	2297 (100.0)	82 (100.0)		
	Medication (mean ± std)	6.62 ± 4.10	3.87 ± 2.97	7.52	**<0.001**
	Sex (*n* females, % females)	1490 (64.9)	50 (61.0)	0.37	0.543
	Age (mean ± std)	75.54 ± 10.94	71.60 ± 6.85	4.99	**<0.001**
<5 medications	Sample size (%)	790 (34.4)	57 (69.5)		
	Medication (mean ± std)	2.51 ± 1.30	2.21 ± 1.35	1.44	0.281
	Sex (*n* females, % females)	500 (63.3)	37 (64.9)	0.01	0.917
	Age (mean ± std)	72.54 ± 10.94	71.24 ± 7.04	1.08	0.281
Polypharmacy (≥5 medications)	Sample size (%)	1507 (65.6)	25 (30.5)		
	Medication (mean ± std)	8.75 ± 3.38	7.64 ± 2.04	1.98	0.058
	Sex (*n* females, % females)	990 (65.7)	13 (52.0)	1.48	0.223
	Age (mean ± std)	77.21 ± 10.20	72.42 ± 6.46	3.63	**0.001**
Hyperpolypharmacy (≥10 medications)	Sample size (%)	456 (19.9)	3 (3.7)		
	Medication (mean ± std)	12.61 ± 2.84	11.33 ± 1.53	1.11	NA
	Sex (*n* females, % females)	292 (64.0)	3 (100.0)	0.00	NA
	Age (mean ± std)	77.93 ± 9.96	69.40 ± 1.83	7.40	NA

## 4 DISCUSSION

Patients referred to memory units commonly present with highly complex PP profiles. These individuals are typically older adults with multiple chronic comorbidities – including cardiovascular disease, metabolic disorders, chronic pain syndromes, and psychiatric conditions – requiring long‐term pharmacological treatment. Consequently, it is not uncommon for them to be prescribed five or more medications concurrently, often by different healthcare providers and across various levels of care. In addition to medications targeting somatic conditions, many patients are also treated with psychotropic agents, including antipsychotics, benzodiazepines, and antidepressants. The use of these drugs, particularly in older populations, is of special concern due to their potential cognitive and sedative effects, increased risk of falls, and association with adverse events. Moreover, cumulative anticholinergic burden, drug–drug interactions, and prescribing cascades may further complicate the clinical picture.

PP has been shown to carry a substantial economic burden for older adults and the health system^27^ and has been described as a risk factor for mortality and hospitalization.^28^ This substantial pharmacological complexity represents a critical challenge in the assessment of cognitive impairment. Medication‐related cognitive side effects may mimic, exacerbate, or obscure underlying neurodegenerative processes.

In this study, the PP profile described reflects the patients’ baseline medication status at the time of referral to the memory unit. This large real‐world study demonstrates that PP and exposure to cognitively deleterious and potentially inappropriate medications are highly prevalent among patients attending a specialized memory clinic. PP represents a systemic prescribing challenge within cognitively vulnerable populations. The observed rates exceed those typically reported in general older adult populations, underscoring the clinical complexity of individuals evaluated for cognitive impairment and dementia. The high prevalence of benzodiazepines, antipsychotics, antidepressants, and anticholinergic medications is particularly concerning given their established association with cognitive decline and dementia risk. Higher exposure to CNS‐active medications was observed in participants with greater cognitive impairment, although the directionality of this relationship cannot be established. These findings emphasize the importance of regular medication review and deprescribing strategies. Furthermore, given that renal function declines with age and influences drug clearance, these results reinforce the need for systematic renal function assessment in older patients with multiple medications. Careful dose adjustment and medication review are therefore essential to minimize drug‐related harm in this high‐risk group.

Memory units implement multidomain, person‐centered intervention plans aimed at slowing cognitive decline, preserving functional autonomy, and optimizing overall brain health. These programs combine structured cognitive stimulation, individualized cognitive training, and goal‐oriented cognitive rehabilitation to enhance memory, executive function, attention, and daily functioning. Supervised physical exercise – including aerobic, resistance, balance, and dual‐task training – is systematically incorporated to promote neuroplasticity, improve cerebral perfusion, and reduce frailty. Rigorous cardiovascular risk control targeting hypertension, diabetes, dyslipidemia, obesity, smoking, and sedentary behavior is integrated due to its well‐established impact on cognitive trajectories. Nutritional interventions are also central, typically promoting adherence to Mediterranean or MIND dietary patterns rich in fruits, vegetables, whole grains, legumes, olive oil, nuts, and fish, while reducing ultra‐processed foods and saturated fats. When clinically indicated, supplementation with vitamin D, vitamin B12, folate, or omega‐3 fatty acids is considered, particularly in cases of documented deficiency or malnutrition risk. Additional components include sleep optimization, psychoeducation, caregiver support, and structured management of neuropsychiatric symptoms. Importantly, comprehensive medication review is routinely conducted to reduce anticholinergic burden, prevent drug–drug interactions, and address inappropriate prescriptions. PP revision and DICI‐based deprescription protocols are implemented as standard elements of care, ensuring pharmacological optimization and minimizing treatments that may adversely affect cognition and functional status.

A key strength of this study is the systematic and scalable extraction of medication data using a LLM applied to structured and semi‐structured clinical records. In routine memory clinic settings, medication data are often recorded in heterogeneous formats that limit large‐scale analysis. By automating extraction and ATC coding with high concordance, we enabled reproducible and comprehensive medication profiling across a large cohort. Such methodological innovation addresses a common bottleneck in pharmacoepidemiologic research, where medication reconciliation is frequently manual, time‐consuming, and inconsistently reported. The high agreement metrics observed support the reliability of this approach and demonstrate the feasibility of integrating artificial intelligence into clinical research workflows. A LLM‐based system could facilitate the identification of patients exhibiting higher DICI or PIM therapeutic patterns, who may be at increased risk of medication‐related complications and therefore could especially benefit from a comprehensive medication review and closer clinical monitoring. From a clinical perspective, our results reinforce the need for systematic medication review as a core component of memory clinic practice. Given the high prevalence of benzodiazepines and anticholinergic drugs, structured deprescribing strategies should be considered, particularly when cognitive complaints or acetylcholinesterase inhibitors are present. Medication optimization may reduce adverse drug events and improve overall treatment safety in older adults with cognitive vulnerability. While not all exposure implies causality, reducing unnecessary CNS‐active medications could improve symptom burden and reduce adverse outcomes.

In the CSF subsample, Aβ‐positive individuals showed significantly lower use of anticholinergic drugs, antidepressants, and benzodiazepines compared with Aβ‐negative participants, while no differences were observed for antipsychotics or other psychotropic medications. Medication analysis was conducted during the initial cognitive evaluation, prior to the availability of CSF biomarker results, and thus the higher prevalence of these medications in Aβ‐negative individuals could suggest a greater contribution of potentially reversible or non‐AD‐related factors – such as psychiatric or sleep disorders – to their cognitive symptoms. Overall, these differences underscore the importance of systematically reviewing medication profiles in memory clinic populations, as certain drug classes may exacerbate cognitive impairment and contribute to diagnostic complexity.

Clinical trial participants showed a lower prevalence of PP and drugs associated with DICI. These differences likely reflect both stricter eligibility criteria and clinical efforts to minimize confounding factors that could interfere with cognitive outcomes. These findings emphasize that PP – and particularly exposure to medications with CNS effects – should be carefully considered in both clinical practice and the design and interpretation of AD clinical trials. Real‐world patients typically present with a higher burden of comorbidities and concomitant medications compared with participants enrolled in clinical trials, who are often selected based on strict inclusion and exclusion criteria that limit PP, the use of potentially interacting drugs, and long washout periods. As a result, trial populations tend to have lower medication complexity and reduced exposure to drugs associated with adverse effects or pharmacodynamic interactions. Trial populations are intentionally selected using strict inclusion and exclusion criteria to reduce clinical heterogeneity, improve safety, maximize internal validity, and minimize potential confounding factors. Consequently, the external validity of trial findings may be limited, underscoring the need for post‐marketing and real‐world evidence studies to better understand how concomitant medication burden influences safety, tolerability, and overall treatment effectiveness in routine clinical practice. Our results have important implications for the implementation of new AD disease‐modifying therapies. PP may affect treatment safety and efficacy, reinforcing the need for integrated pharmacological assessment in memory clinics.

These findings should be considered within the context of the study design. An important interpretational limitation is the inability to distinguish whether medication burden contributes to cognitive impairment or instead reflects disease severity. Therefore, PP and DICI exposure should primarily be interpreted as markers of clinical complexity rather than direct causal determinants of cognitive decline. The study did not include adjustment for comorbidity burden or neuropsychiatric symptom severity, both of which are likely major determinants of medication exposure. Future longitudinal approaches incorporating repeated assessments, treatment modifications, and time‐to‐event modeling, ideally complemented by pharmacogenetic and biomarker data, may better clarify whether reducing inappropriate medication exposure modifies clinical trajectories.

Even so, the consistency of the associations across a large, well‐characterized cohort supports the relevance of the observations. The combination of scale, biomarker characterization, and automated medication extraction enhances robustness and illustrates how advanced computational methods can be integrated into the study of PP in complex clinical populations while maintaining methodological transparency.

Furthermore, including pre‐emptive pharmacogenetic testing can improve psychotropic prescribing by identifying genetic variants that affect drug metabolism and treatment response. For antidepressants, CYP2D6 and CYP2C19 polymorphisms strongly influence drug exposure, efficacy, and adverse effects. For antipsychotics, CYP2D6, CYP1A2, and CYP3A4 variability can affect metabolism, tolerability, and outcomes. In older adults with cognitive impairment, who often experience PP and age‐related physiological changes, combining pharmacogenetic data with renal function monitoring is especially important, as reduced renal clearance may increase toxicity, drug interactions, and adverse outcomes. Integrating both approaches could enable safer, more individualized psychotropic treatment in this vulnerable population.

In conclusion, PP and exposure to cognitively deleterious medications are highly prevalent among patients evaluated for cognitive impairment. Increasing medication burden is consistently associated with higher exposure to inappropriate drugs, independent of age group. The use of automated LLM‐based medication extraction represents an innovative and scalable methodological advance that enables systematic investigation of prescribing patterns in real‐world clinical practice. These findings support the urgent need for structured medication review and deprescribing strategies as part of comprehensive dementia care.

## CONFLICT OF INTEREST STATEMENT

The authors declare no conflicts of interest. Author disclosures are available in the Supporting Information.

## CONSENT STATEMENT

All human subjects provided informed consent.

## Supporting information




**Supporting material**: alz71708‐sup‐0001‐SuppMat.docx

**Supporting material**: alz71708‐supp‐0002‐ICMJE.pdf
