# Supplementary material for: When treatment becomes risk: polypharmacy and inappropriate prescribing in cognitive impairment in 2379 consecutive patients from a memory clinic
Source: Alzheimers Dement. 2026 Aug 25;22(8):e71708. doi: 10.1002/alz.71708 (PMC13504620; doi:10.1002/alz.71708)
Supplement: Supplementary file 2 [file ALZ-22-e71708-s002.docx]

**When Treatment Becomes Risk: Polypharmacy and Inappropriate Prescribing in Cognitive Impairment in 2,379 consecutive patients from a Memory Clinic**

# Xavier Morató ^1,2*^, Adrian Hinojosa-Calleja^1*^, Sara Jofresa^1^, Berta Calm^1^, Amanda Cano^1,2^, Josep Blazquez-Folch^1^, Emilio Alarcón-Martín^1^, Laura Montrreal^1^, Álvaro Muñoz-Morales^1^, Claudia Olivé^1^, Paula Bayón^1^, Oscar Sotolongo-Grau^1^, Adelina Orellana ^1^, Pilar Sanz-Cartagena^1^, Xavier Montalban^1,2^, Marta Marquié^1,2^, Maria Victoria Fernandez ^1,2^, Agustín Ruiz^1,2,3,4^, Mercè Boada^1,2^, Sergi Valero^1,2^

^1^Ace Alzheimer Center Barcelona - Universitat Internacional de Catalunya, Barcelona, Spain.

^2^Networking Research Center on Neurodegenerative Diseases (CIBERNED), Instituto de Salud Carlos III, Madrid, Spain.

^3^Glenn Biggs Institute for Alzheimer's & Neurodegenerative Diseases, University of Texas Health Science Center. 8300 Floyd Curl Drive, San Antonio, TX 78229, USA.

^4^Department of Microbiology, Immunology and Molecular Genetics, Long School of Medicine. University of Texas Health Science Center. 7703 Floyd Curl Drive, San Antonio, TX 78229, USA.

*Authors contributed equally to this work

**Supplementary Tables**

**Supplementary Table 1:** List of drugs with anticholinergic properties

| ALIMEMAZINE (R06AD01) | KETOROLAC (S01BC05,M01AB15) |
| --- | --- |
| AMANTADINE (N04BB01) | KETOTIFEN (S01GX08,R06AX17) |
| AMITRIPTYLINE (N06AA09,N06CA01) | LITHIUM (N05AN01) |
| ATROPINE (A03BA01,S01FA01) | LEVOMEPROMAZINE (N05AA02) |
| BACLOFEN (M03BX01) | LOPERAMIDE (A07DA03,A07DA53) |
| BIPERIDEN (N04AA02) | LORATADINE (R06AX13) |
| BROMPHENIRAMINE (R06AB01) | MECLOZINE (R06AE05) |
| BROMOCRIPTINE (G02CB01,N04BC01) | MELITRACEN (N06CA02) |
| BUTYLSCOPOLAMINE (A03BB01,A03DB04) | MEPERIDINE (N02AB02) |
| CARBAMAZEPINE (N03AF01) | METHADONE (N07BC02) |
| CETIRIZINE (R06AE07) | METHOCARBAMOL (M03BA03,M03BA53) |
| CYCLOBENZAPRINE (M03BX08) | MIRTAZAPINE (N06AX11) |
| CYPROHEPTADINE (R06AX02) | MORPHINE (A07DA02,N02AA01) |
| CITALOPRAM (N06AB04) | NORTRIPTYLINE (N06AA10) |
| CLOMIPRAMINE (N06AA04) | OLANZAPINE (N05AH03) |
| CLONAZEPAM (N03AE01) | OPICAPONE (N04BX04) |
| CHLORDIAZEPOXIDE (N05BA02) | OXCARBAZEPINE (N03AF02) |
| CHLORPHENIRAMINE (R01BA53,H02BX93,R01AB) | OXYBUTYNIN (G04BD04) |
| CHLORPROMAZINE (N05AA01) | OXYCODONE (N02AA05,N02AA55) |
| CLOZAPINE (N05AH02) | PALIPERIDONE (N05AX13) |
| CLOCINIZINE, PHENYLPROPANOLAMINE (R06AE92) | PAROXETINE (N06AB05) |
| CODEINE (R05DA04,N02AJ06,N02AJ07,N02AJ08,R05DA20,N02AA79) | PERPHENAZINE (N05AB03) |
| DESFESORETODINE (G04BD13) | PIMOZIDE (N05AG02) |
| DESLORATADINE (R06AX27,R01BA52) | MEPIRAMINA (R03DA12) |
| DEXCLORPHENIRAMINE (R06AB02) | PRIDINOL (M03BX03) |
| DIAZEPAM (N05BA01) | PROCYCLIDINE (N04AA04) |
| DICYCLOVERINE (A03AA07,M04AC51) | PROMETHAZINE (D04AA10,R05CB10) |
| DIPHENYDRAMINE (D04AA32,N05CM,R06AA02,R06AA03,R06AA52) | PROPIVERINE (G04BD06) |
| DIMENHYDRINATE (R06AA11) | QUETIAPINE (N05AH,N05AH04) |
| DISOPYRAMIDE (C01BA03) | RANITIDINE (A02BA02) |
| DOMPERIDONE (A03FA03) | RISPERIDONE (N05AX08) |
| DOXEPIN (N06AA12) | SOLIFENACIN (G04BD08,G04CA53) |
| DOXYLAMINE (R06AA09,R06AA59) | TEMAZEPAM (N05CD07) |
| ENTACAPONE (N04BX02,N04BA03) | THEOPHYLINE (R03DA04) |
| SCOPOLAMINE (A04AD01) | TIZANIDINE (M03BX02) |
| FENTANYL (N02AB03) | TOLCAPONE (N04BX01) |
| FESOTERODINE (G04BD11) | TOLTERODINE (G04BD07) |
| FEXOFENADINE (R06AX26) | TRAMADOL (N02AX02,N02AJ13,N02AJ14,N02AJ16) |
| FLAVOXATE (G04BD02) | TRAZODONE (N06AX05) |
| FLUOXETINE (N06AB03) | TRIAZOLAM (N05CD05) |
| FLUVOXAMINE (N06AB08) | TRIHEYPHENIDYL (N04AA01) |
| HALOPERIDOL (N05AD01) | TRIMIPRAMINE (N06AA06) |
| HYDROXYZINE (N05BB01) | TROSPIUM (G04BD09) |
| IMIPRAMINE (N06AA02) |  |

**Supplementary Table 2:** Performance Metrics of DICI and PIM Classification in the Validation Sample

|  | **ACC** | **SEN** | **SPE** |
| --- | --- | --- | --- |
| **DICI** | 97.44(92.81,99.04) | 96.3(89.67,98.73) | 100.0(90.36,100) |
| **PIM** | 100.0(96.86,100) | 100.0(87.94,100) | 100.0(95.86,100) |
| **Anticholinergic** | 96.58(91.63,98.06) | 92.59(82.45,97.08) | 100.0(94.25,100.0) |
| **Benzodiazepines** | 97.44(92.81,99.04) | 92.31(79.68,97.35) | 100.0(95.31,100) |
| **Hypnotics** | 100.0(96.86,100) | 100.0(34.24,100) | 100.0(96.77,100.0) |
| **Antipsychotics** | 99.15(95.37,99.79) | 93.75(71.67,98.89) | 100.0(96.34,100.0) |
| **Opioids** | 97.44(92.81,99.04) | 80.0(54.81,92.95) | 100.0(96.37,100) |
| **Antidepressants** | 100.0(96.86,100) | 100.0(89.57,100) | 100.0(95.63,100.0) |
| **Antiepileptic** | 100.0(96.86,100) | 100.0(51.01,100) | 100.0(96.71,100) |
| **Dopaminergic** | 100.0(96.86,100) | 100.0(51.01,100) | 100.0(96.71,100) |
| **Glucocorticoids** | 100.0(96.86,100) | 100.0(34.24,100) | 100.0(96.77,100.0) |

ACC: Accuracy, SEN: Sensitivity, SPE: Specificity

**Supplementary Table 3:** Prevalence of PP and HP in patients with cognitive impairment

| **Group** | **Sample size** | **CDR 0** | **CDR 0.5** | **CDR 1** | **CDR 2** | **CDR 3 (n, %)** |
| --- | --- | --- | --- | --- | --- | --- |
|  |  | **(n, %)** | **(n, %)** | **(n, %)** | **(n, %)** |  |
| **Whole population** | | | | | | |
| Whole sample size | 2362 | 251 (10.6%) | 1014 (42.9%) | 708 (30.0%) | 336 (14.2%) | 45 (1.9%) |
| <5 meds | 843 | 148 (17.6%) | 395 (46.9%) | 206 (24.4%) | 84 (10.0%) | 8 (0.9%) |
| Polypharmacy (≥5 meds) | 1519 | 103 (6.8%) | 619 (40.8%) | 502 (33.0%) | 252 (16.6%) | 37 (2.4%) |
| Hyperpolypharmacy (≥10 meds) | 455 | 21 (4.6%) | 167 (36.7%) | 157 (34.5%) | 94 (20.7%) | 12 (2.6%) |
| **Age < 75** | | | | | | |
| Whole sample size | 963 | 219 (22.7%) | 537 (55.8%) | 153 (15.9%) | 46 (4.8%) | 6 (0.6%) |
| <5 meds | 455 | 132 (29.0%) | 242 (53.2%) | 63 (13.8%) | 15 (3.3%) | 2 (0.4%) |
| Polypharmacy (≥5 meds) | 508 | 87 (17.1%) | 295 (58.1%) | 90 (17.7%) | 31 (6.1%) | 4 (0.8%) |
| Hyperpolypharmacy (≥10 meds) | 143 | 18 (12.6%) | 89 (62.2%) | 23 (16.1%) | 10 (7.0%) | 2 (1.4%) |
| **Age ≥ 75** | | | | | | |
| Whole sample size | 1399 | 32 (2.3%) | 477 (34.1%) | 555 (39.7%) | 290 (20.7%) | 39 (2.8%) |
| <5 meds | 388 | 16 (4.1%) | 153 (39.4%) | 143 (36.9%) | 69 (17.8%) | 6 (1.5%) |
| Polypharmacy (≥5 meds) | 1011 | 16 (1.6%) | 324 (32.0%) | 412 (40.8%) | 221 (21.9%) | 33 (3.3%) |
| Hyperpolypharmacy (≥10 meds) | 312 | 3 (1.0%) | 78 (25.0%) | 134 (42.9%) | 84 (26.9%) | 10 (3.2%) |

**Supplementary Table 4:** Number of drugs associated with DICI (1-5) in the whole population

| **Group** | **Sample size** | **1** | **2** | **3** | **4** | **5** |
| --- | --- | --- | --- | --- | --- | --- |
| **Whole population** | | | | |  |  |
| Whole sample size | 2379 | 510 (21.4) | 528 (22.2) | 312 (13.1) | 81 (3.4) | 12 (0.5) |
| <5 meds | 847 | 160 (18.9) | 119 (14.0) | 35 (4.1) | 5 (0.6) | 3 (0.4) |
| Polypharmacy  (≥5 meds) | 1532 | 350 (22.8) | 409 (26.7) | 277 (18.1) | 76 (5.0) | 9 (0.6) |
| Hyperpolypharmacy (≥10 meds) | 459 | 93 (20.3) | 135 (29.4) | 123 (26.8) | 43 (9.4) | 6 (1.3) |
| **Age < 75** | | | | |  |  |
| Whole sample size | 969 | 179 (18.5) | 217 (22.4) | 129 (13.3) | 35 (3.6) | 8 (0.8) |
| <5 meds | 457 | 82 (17.9) | 62 (13.6) | 25 (5.5) | 4 (0.9) | 3 (0.7) |
| Polypharmacy  (≥5 meds) | 512 | 97 (18.9) | 155 (30.3) | 104 (20.3) | 31 (6.1) | 5 (1.0) |
| Hyperpolypharmacy (≥10 meds) | 144 | 21 (14.6) | 46 (31.9) | 43 (29.9) | 18 (12.5) | 3 (2.1) |
| **Age ≥ 75** | | | | |  |  |
| Whole sample size | 1410 | 331 (23.5) | 311 (22.1) | 183 (13.0) | 46 (3.3) | 4 (0.3) |
| <5 meds | 390 | 78 (20.0) | 57 (14.6) | 10 (2.6) | 1 (0.3) | 0 (0.0) |
| Polypharmacy  (≥5 meds) | 1020 | 253 (24.8) | 254 (24.9) | 173 (17.0) | 45 (4.4) | 4 (0.4) |
| Hyperpolypharmacy (≥10 meds) | 315 | 72 (22.9) | 89 (28.3) | 80 (25.4) | 25 (7.9) | 3 (1.0) |

**Supplementary Table 5:** List of Active principle and ATC code of the most common prescribed DICI in the whole population

| **Drug group** | **Medication** | **n (%)** |
| --- | --- | --- |
| DICI | LORAZEPAM (N05BA06) | 317 (13.3) |
|  | QUETIAPINE (N05AH04) | 234 (9.8) |
|  | SERTRALINE (N06AB06) | 278 (11.7) |
|  | TRAZODONE (N06AX05) | 163 (6.9) |
|  | CITALOPRAM (N06AB04) | 152 (6.4) |
|  | DIAZEPAM (N05BA01) | 122 (5.1) |
|  | TRAMADOL AND PARACETAMOL (N02AJ13) | 109 (4.6) |
|  | ALPRAZOLAM (N05BA12) | 99 (4.2) |
|  | TRAMADOL (N02AX02) | 78 (3.3) |
|  | PREDNISONE (H02AB07) | 67 (2.8) |
| Benzodiazepines | LORAZEPAM (N05BA06) | 317 (13.3) |
|  | DIAZEPAM (N05BA01) | 122 (5.1) |
|  | LORMETAZEPAM (N05CD06) | 103 (4.3) |
|  | ALPRAZOLAM (N05BA12) | 99 (4.2) |
|  | ZOLPIDEM (N05CF02) | 45 (1.9) |
| Antipsychotics | QUETIAPINE (N05AH04) | 234 (9.8) |
|  | RISPERIDONE (N05AX08) | 56 (2.4) |
|  | OLANZAPINE (N05AH03) | 29 (1.2) |
|  | ARIPIPRAZOLE (N05AX12) | 22 (0.9) |
|  | LEVOSULPIRIDE (N05AL07) | 15 (0.6) |
| Opioids | TRAMADOL AND PARACETAMOL (N02AJ13) | 109 (4.6) |
|  | TRAMADOL (N02AX02) | 78 (3.3) |
|  | FENTANYL (N02AB03) | 27 (1.1) |
|  | TAPENTADOL (N02AX06) | 26 (1.1) |
|  | CODEINE AND PARACETAMOL (N02AJ06) | 4 (0.2) |
| Antidepressants | SERTRALINE (N06AB06) | 278 (11.7) |
|  | CITALOPRAM (N06AB04) | 152 (6.4) |
|  | FLUOXETINE (N06AB03) | 65 (2.7) |
|  | ESCITALOPRAM (N06AB10) | 48 (2.0) |
|  | PAROXETINE (N06AB05) | 46 (1.9) |
| Antiepileptic | CLONAZEPAM (N03AE01) | 57 (2.4) |
|  | LEVETIRACETAM (N03AX14) | 23 (1.0) |
|  | LAMOTRIGINE (N03AX09) | 16 (0.7) |
|  | TOPIRAMATE (N03AX11) | 16 (0.7) |
|  | GABAPENTIN (N03AX12) | 11 (0.5) |
| Dopaminergic | LEVODOPA/DECARBOXYLASE (N04BA02) | 40 (1.7) |
|  | PRAMIPEXOLE (N04BC05) | 14 (0.6) |
|  | RASAGILINE (N04BD02) | 6 (0.3) |
|  | ROPINIROLE (N04BC04) | 3 (0.1) |
|  | AMANTADINE (N04BB01) | 3 (0.1) |
| Glucocorticoids | PREDNISONE (H02AB07) | 67 (2.8) |
|  | DEXAMETHASONE (H02AB02) | 14 (0.6) |
|  | METHYLPREDNISOLONE (H02AB04) | 8 (0.3) |
|  | HYDROCORTISONE (H02AB09) | 3 (0.1) |
|  | DEFLAZACORT (H02AB13) | 2 (0.1) |
| Hypnotics | ZOLPIDEM (N05CF02) | 45 (1.9) |
| Anticholinergics | QUETIAPINE (N05AH04) | 234 (9.8) |
|  | TRAZODONE (N06AX05) | 163 (6.9) |
|  | CITALOPRAM (N06AB04) | 152 (6.4) |
|  | DIAZEPAM (N05BA01) | 122 (5.1) |
|  | TRAMADOL AND PARACETAMOL (N02AJ13) | 109 (4.6) |

**Supplementary Table 6:** DICI burden according to CDR category, overall and stratified by age group

| Whole population | | | | | | | |
| --- | --- | --- | --- | --- | --- | --- | --- |
|  | **CDR 0** | **CDR 0.5** | **CDR 1** | **CDR 2** | **CDR 3** | **Spearman rho** | **p-value** |
| **DICI burden** | 1.1 ± 1.4 | 1.3 ± 1.4 | 1.3 ± 1.3 | 1.7 ± 1.4 | 1.9 ± 1.3 | 0.124 | <.001 |

| Age < 75 | | | | | | | |
| --- | --- | --- | --- | --- | --- | --- | --- |
| **DICI burden** | **CDR 0** | **CDR 0.5** | **CDR 1** | **CDR 2** | **CDR 3** | **Spearman rho** | **p-value** |
| **DICI burden** | 1.1 ± 1.4 | 1.4 ± 1.5 | 1.4 ± 1.5 | 1.9 ± 1.7 | 2.3 ± 1.6 | 0.118 | <.001 |

| Age ≥75 | | | | | | | |
| --- | --- | --- | --- | --- | --- | --- | --- |
|  | **CDR 0** | **CDR 0.5** | **CDR 1** | **CDR 2** | **CDR 3** | **Spearman rho** | **p-value** |
| **DICI burden** | 0.9 ± 1.2 | 1.1 ± 1.2 | 1.3 ± 1.3 | 1.6 ± 1.3 | 1.8 ± 1.2 | 0.165 | <.001 |

**Supplementary Table 7:** Anticholinergic and Antipsychotic Exposure Primarily Drives the Association Between DICI Burden and Cognitive Severity

|  | **Model 1** | | **Model 2** | | **Model 3** | |
| --- | --- | --- | --- | --- | --- | --- |
| **AIC** | 5121 | | 5155 | | 5107 | |
| **BIC** | 5167 | | 5201 | | 5153 | |
|  | **OR (95% CI)** | **p-value** | **OR (95% CI)** | **p-value** | **OR (95% CI)** | **p-value** |
| **DICI burden** | 1.057 (1.022, 1.093) | **<0.001** |  |  |  |  |
| **DICI - (AC+AP)**  **burden** |  |  | 1.026 (0.976,1.078) | 0.318 |  |  |
| **AC +AP**  **burden** |  |  |  |  | 1.080 (1.014,1.151) | **0.017** |
| **Age** | 1.058 (1.053, 1.064) | **<0.001** | 1.057 (1.052,1.063) | **<0.001** | 1.056 (1.051,1.062) | **<0.001** |
| **Years of education** | 0.929 (-0.920,0.938) | **<0.001** | 0.933 (0.924,0.943) | **<0.001** | 0.924 (0.915,0.933) | **<0.001** |
| **Sex (Female)** | 0.970 (0.882,1.067) | 0.534 | 1.016 (0.923, 1.119) | 0.745 | 0.908 (0.825,0.999) | **0.047** |

**Supplementary Table 8:** Adjusted Logistic Regression Models Evaluating Medication Exposure by Amyloid-β Status

|  | **OR AB+ vs AB− (95% CI)** | **p-value** |
| --- | --- | --- |
| **DICI** | 0.621 (0.420-0.920) | **0.017** |
| **Benzodiazepines** | 0.678 (0.444-1.033) | 0.071 |
| **Antipsychotics** | 0.582 (0.294-1.152) | 0.120 |
| **Opioids** | 0.536 (0.282-1.019) | 0.057 |
| **Hypnotics** | 1.079 (0.263-4.431) | 0.916 |
| **Antidepressants** | 1.118 (0.724-1.724) | 0.615 |
| **Antiepileptic** | 0.469 (0.190-1.156) | 0.100 |
| **Dopaminergic** | 0.665 (0.126-3.504) | 0.630 |
| **Glucocorticoids** | 0.704 (0.206-2.403) | 0.576 |
| **Antticholinergics** | 0.565 (0.383-0.834) | **0.004** |
